# Remodeling of Energy Metabolism and Absence of Electrophysiological Changes in the Heart of Obese Hyperleptinemic Mice. New Insights into the Pleiotropic Role of Leptin

**DOI:** 10.3389/fendo.2013.00175

**Published:** 2013-11-15

**Authors:** Rocío Guzmán-Ruiz, Nieves Gómez-Hurtado, Marta Gil-Ortega, Beatriz Somoza, M. Carmen González, Isabel Aránguez, Miriam Martín-Ramos, Carmen González-Martín, Carmen Delgado, Marisol Fernández-Alfonso, Mariano Ruiz-Gayo

**Affiliations:** ^1^Departamento de Ciencias Farmacéuticas y de la Salud, Facultad de Farmacia, Universidad CEU-San Pablo, Madrid, Spain; ^2^Departamento de Farmacología. Facultad de Medicina, Universidad Complutense, Madrid, Spain; ^3^Departamento de Fisiología, Facultad de Medicina, Universidad Autónoma, Madrid, Spain; ^4^Departamento de Bioquímica, Facultad de Farmacia, Universidad Complutense, Madrid, Spain; ^5^Centro de Investigaciones Biológicas, Consejo Superior de Investigaciones Científicas, Madrid, Spain; ^6^Instituto Pluridisciplinar-Departamento de Farmacología, Facultad de Farmacia, Universidad Complutense, Madrid, Spain

**Keywords:** leptin, cardiac metabolism, UCP3, electrophysiology, obesity

## Abstract

Dietary treatment with high-fat diets (HFD) triggers diabetes and hyperleptinemia, concomitantly with a partial state of leptin resistance that affects hepatic and adipose tissue but not the heart. In this context, characterized by widespread steatosis, cardiac lipid content remains unchanged. As previously reported, HFD-evoked hyperleptinemia could be a pivotal element contributing to increase fatty-acid (FA) metabolism in the heart and to prevent cardiac steatosis. This metabolic adaptation might theoretically reduce energy efficiency in cardiomyocytes and lead to cardiac electrophysiological remodeling. Therefore the aim of the current study has been to investigate the impact of long-term HFD on cardiac metabolism and electrophysiological properties of the principal ionic currents responsible of the action potential duration in mouse cardiomyocytes. Male C57BL/6J mice were fed a control (10 kcal% from fat) or HFD (45 kcal% from fat) during 32 weeks. Quantification of enzymatic activities regulating mitochondrial uptake of pyruvate and FA showed an increase of both carnitine-palmitoyltransferase and citrate synthase activities together with a decrease of lactate dehydrogenase and pyruvate dehydrogenase activities. Increased expression of uncoupling protein-3, Mn-, and Cu/Zn-superoxide dismutases and catalase were also detected. Total glutathione/oxidized glutathione ratios were unaffected by HFD. These data suggest that HFD triggers adaptive mechanisms aimed at (i) facilitating FA catabolism, and (ii) preventing oxidative stress. All these changes did not affect the duration of action potentials in cardiomyocytes and only slightly modified electrocardiographic parameters.

## Introduction

Obesity develops in a complex scenario characterized by an altered adipokine profile associated to systemic low-grade inflammation and, often, to diabetes (1, 2). There is now compelling evidence that an altered pattern of adipokine release together with insulin resistance are main causes of heart metabolism remodeling and cardiac disease in obese individuals (3–6). Nevertheless the fact that insulin resistance is not systematically present in obese individuals makes difficult to identify the link between obesity and cardiomyopathy (2, 7). Previous research of our group has evidenced the up-regulation of enzymatic activities involved in fatty-acid (FA) catabolism in cardiac tissue of diet-induced obese (DIO) mice (8, 9). These metabolic changes might be linked to a particular endocrine environment characterized by hyperleptinemia and be protective for cardiac tissue as they might account for the lack of cardiac steatosis observed in these animals (10, 11). It has to be noted that the experimental model used in these former studies is based on an eight-month HFD treatment schedule in mice that were 5-week-old when treatment started, and deals with elevated HOMA-IR indexes and widespread leptin resistance that affects most tissues except the heart. Interestingly, we have observed that a high HOMA-IR index is an insufficient condition to cause hepatic steatosis in absence of hepatic resistance to leptin (10).

Although FA are the main source of energy in cardiac tissue, an overload of dietary fat has been shown to promote mitochondrial β-oxidation to the detriment of pyruvate catabolism. FA oxidation is less efficient than glucose/pyruvate in terms of energy production because FA consume more oxygen than glucose does (12–14) and they can be ejected from mitochondria by means of the uncoupling protein-3 (UCP3), leading to a mitochondria-cytosol futile FA cycling (15). Otherwise, excessive FA oxidation has been shown to increase citrate production which can inhibit phosphofructokinase, a key enzyme recruiting fructose-6-phosphate into the glycolytic pathway, and also impair pyruvate oxidation (16–18). Finally, under these conditions, the rate of reactive oxygen species (ROS) production can be increased (19), and cause mitochondrial damage (20). In fact, optimizing energy metabolism by increasing glucose oxidation has been proposed to be an useful approach to prevent and treat cardiac dysfunction in obese people (15) as well as a valuable therapeutic intervention in failing hearts (3, 21, 22).

The aim of the current study was to further characterize the influence of long-term DIO on cardiac metabolism and the eventual association of metabolic remodeling and cardiac electrophysiological properties. For this purpose, we have analyzed in hearts of DIO mice: (i) enzymatic activities of lactate dehydrogenase, pyruvate dehydrogenase, carnitine-palmitoyltransferase (CPT), and citrate synthase (CS), as well as lactate content, (ii) mitochondrial uncoupling, (iii) redox status, (iv) electrophysiological properties of ionic currents responsible for the action potential duration (APD) in cardiomyocytes, and (v) electrocardiographic characteristics.

## Materials and Methods

### Experimental design

Five-week old male C57BL/6J mice (Harlan, Spain) were housed (five per cage) under a 12 h light/12 h dark cycle, in a temperature-controlled room (22°C) with food and water *ad libitum*. Animals were divided in two groups with similar average BW and assigned either to a control or to a high-fat diet (HFD). Control diet (D12450B, 10 kcal% fat, 70 kcal% carbohydrates, and 20 kcal% protein; 3.85 kcal/g) and HFD (D12451, 45 kcal% fat, 35 kcal% carbohydrates, and 20 kcal% protein; 4.73 kcal/g) were supplied by Test Diet Limited BCM IPS Ltd (UK). After 32 week dietary treatment, groups of animals were separated for biochemical and functional studies. The investigation conforms to the Guide for the Care and Use of Laboratory Animals published by the US National Institute of Health (NIH publication No. 85–23, revised 1996). This study was approved by the ethics committee of the University CEU-San Pablo (SAF 2009-09714).

### Plasma biochemistry

Animals were killed by decapitation between 9 and 10 a.m., blood collected in chilled EDTA-coated polypropylene tubes and left ventricle dissected and frozen. Plasma samples were stored at −80°C until assay. For enzymatic activities, tissues were immediately processed and prepared for assay. Proteins, lactate, and glutathione were determined in frozen tissues. Plasma leptin (Linco Research, USA; 4.9% intra-assay variation, 3.3% inter-assay variation) and insulin (Mercodia, Denmark; 2.2% intra-assay variation, 4.9% inter-assay variation) were quantified by specific radioimmunoassay and ELISA, respectively.

### Cardiac triglycerides

Triglyceride content in heart was determined as previously described (8). Briefly, 20 mg of wet tissue were homogenized in a mixture containing 40 ml of 2 mM NaCl/20 mM EDTA/50 mM sodium phosphate buffer (pH 7.4) 40 ml of tert-butanol, and 20 ml of Triton X-100/methanol (1/1). Triglycerides were measured with a Sigma diagnostic kit (USA).

### Quantification of proteins by western-blot

We quantified uncoupling protein-1, 2, and 3 (UCP1, UCP2, UCP3, respectively) Mn- and Cu/Zn-superoxide dismutase (Mn-SOD, Cu/Zn-SOD), and catalase. Western-blotting was performed as previously described (8, 9). Briefly, primary antibodies against UCP1, UCP3 (Affinity BioReagent, USA), UCP2 (Santa Cruz Biothecnology, USA), and Mn-SOD, Zn-SOD, and catalase (Sigma, USA) were applied overnight at 4°C at the appropriate dilution. After washing and incubation with appropriate IgG-peroxidase complexes, blots were incubated in commercial enhanced chemiluminescence reagents (ECL, Amersham Bioscence, UK) and exposed to autoradiographic film. Films were scanned using a GS-800 Calibrated Densitometer (Bio-Rad, Spain) and quantified using Quantity One software (Bio-Rad, Spain). Values were normalized with β-actin to account for variations in gel loading.

### Determination of enzymatic activities

Lactate dehydrogenase, CPT, CS, and pyruvate dehydrogenase activities were measured by means of colorimetric methods as described in the literature (8, 9, 23, 24). Glucose-6-phosphate dehydrogenase (G6PD) activity was determined by measuring Δ absorbance at 340 nm (Versamax, Molecular Devices, USA) due to the conversion of NADP^+^ to NADPH by G6PD and 6-phosphogluconate-dehydrogenase (6PGD). For total activity (G6PD and 6PGD), samples (1 mg/ml protein) were incubated in 96-well microplates containing assay buffer (50 mM Tris pH = 8.1, 1 mM MgCl2, 0.2 mM glucose-6-phosphate, 0.2 mM 6-phosphogluconate, and 0.1 mM NADP^+^). For 6PGD activity determination, samples were incubated in absence of glucose-6-phosphate and then G6PD activity theoretically estimated by subtracting 6PGD activity from total activity. Results were expressed as Δ Abs/min/mg protein (25).

### Determination of superoxide anion, glutathione and lactate

Production of superoxide anion ($O_{2}^{-}$) was determined in fresh samples by measuring lucigenin-enhanced chemiluminescence (26, 27). Briefly, heart explants were incubated for 30 min at 37°C in oxygenated 20 mM HEPES buffer (pH = 7.4, 99 mM NaCl, 4.7 mM KCl, 1.9 mM CaCl_2_, 25 mM NaHCO_3_, 1 mM KH_2_PO_4_, 1.2 mM MgSO_4_, 11 mM glucose). Explants, NADPH (1 mmol/l) and lucigenin (5 μmol/l) were then added to tubes containing 1 ml buffer. $O_{2}^{-}$ production was expressed in arbitrary units/mg protein. Lactate concentration was measured as described (8). For glutathione assay, samples were homogenized in phosphate buffer (pH = 6–7) containing EDTA (1 mM) and centrifuged for 15 min (10,000 *g*; 4°C). Supernatants were collected and kept at −20°C until assay. Both reduced (GSH) and oxidized glutathione (GSSG) concentrations were quantified by colorimetric assay by using a commercial kit (Cayman Chemical, USA) following the manufacturer instructions. Data were expressed as (total glutathione)/(GSSG).

### Isolation of ventricular myocytes

Mice were heparinized (1 IU/kg i.p.) and anesthetized with pentobarbital (50 mg/kg). Hearts were rapidly dissected, mounted on a modified Langendorff apparatus and retrogradely perfused through the aorta (2–3 min, 36–37°C) with Ca^2+^-free Tyrode solution (TS; 130 mM NaCl, 5.4 mM KCl, 0.4 mM NaH_2_PO_4_, 0.5 mM MgCl_2_, 25 mM HEPES, 5 mM NaHCO_3_, 22 mM glucose; pH adjusted to 7.4 with NaOH) containing 0.2 mM EGTA, then 3–4 min with TS containing 251 IU/ml type II collagenase (Worthington, USA), and 0.1 mM CaCl_2_. After perfusion, hearts were chopped in small pieces, and gently stirred in TS containing 1 mg/ml bovine serum albumin (BSA, Sigma, USA). Isolated cells were filtered, centrifuged and suspended in TS containing 2 mg/ml BSA and 0.5 mM CaCl_2_. After centrifugation, cells were suspended in a solution containing 1 mM CaCl_2_ and 2 mg/ml BSA and stored at room temperature until electrophysiological assays (within 5 h of the isolation).

### Cellular electrophysiology

Electrophysiological experiments were performed at room temperature (24–26°C) on Ca^2+^-tolerant rod-shaped myocytes. Whole-cell configuration of the patch-clamp technique was employed to measure the L-type Ca^2+^ current (*I*_CaL_), the transient outward K^+^ current (Ito_total_), the ultra-rapid delayed rectifier K^+^ current (*I*_Kur_), and the steady-state outward K^+^ current (*I*_ss_). The voltage-clamp circuit was provided by an Axopatch-200B amplifier controlled by a computer equipped with a pClamp 6.0 and interfaced to the amplifier with a Digidata 1322A (Axon Instruments, USA). Recording pipettes were made from 1.5 mm-OD soft-glass capillary tubing by using a microprocessor-based patch-clamp puller (P97/PC, Sutter Instruments, USA). Tip resistances after filling with the internal solution ranged between 0.9–2 MΩ. Whole-cell currents were expressed as current densities, which were calculated from the current amplitude normalized by the membrane capacitance. Membrane capacitance (*C*_m_) was calculated according to the equation *C*_m_ = τ_c_ *I*_0_/Δ*E*_m_ [1 − (*I*_∞_/*I*_0_)] (τ_c_, time constant of the membrane capacitance; *I*_0_ maximum capacitance current value; Δ*E*_m_, amplitude of the voltage step; *I*∞, amplitude of the steady-state current). Cm was elicited by applying ±10 mV voltage steps from a holding potential of −60 mV. Current-voltage (*I* − *V*) relationship for the *I*_CaL_ was obtained from a holding potential of –50 mV and cell depolarization (300 ms, 0.2 Hz) in 10 mV increments from −40 to +60 mV. Current-voltage (*I* − *V*) relationship for Ito_total_ was constructed from 500 ms series test potentials varying from −70 to +50 mV in 10 mV increments, from a holding potential of −80 mV at a frequency rate of 0.1 Hz. The same protocol was applied but now preceded by a 100 ms inactivating pre-pulse (−40 mV) in order to suppress the transient portion of the Ito_total_ (28). The remaining current (*I*_Kslow_) is composed of both a *I*_Kur_ (4-aminopyridine-sensitive component) and a *I*_ss_ (4-aminopyridine-resistant component). For *I*_ss_ recording, an inactivating pre-pulse was applied in presence of 250 μM 4-AP. *I*_Kur_ was calculated by subtracting *I*_ss_ from the current recorded in the absence 4-AP. Current densities were determined at the peak current. Action potentials (AP) were elicited at 10 s intervals by 1.5-fold excitation threshold current pulses of 2.5 ms in duration. After stabilization of the records, 10 successive APs were recorded. The parameters of the APs for each cell corresponded to the mean of these 10 APs. The APD was measured at 20, 50, and 90% repolarization. The solution for *I*_CaL_ recording contained (in mM): 140 NaCl, 1.1 MgCl_2_, 5.4 CsCl, 10 glucose, 5 HEPES, 1.8 CaCl_2_; pH adjusted to 7.4 with NaOH. The intracellular recording pipette solution for whole-cell experiments contained (in mM): 100 CsCl, 20 triethylamine, 5 EGTA, 10 HEPES, 5 Na_2_ATP, 0.4 Na_2_GTP, 5 Na_2_creatine phosphate, 0.06 CaCl_2_; pH adjusted to 7.4 with CsOH. The solution for K^+^ current recordings contained (in mM): 135 NaCl, 10 glucose, 10 HEPES, 1 MgCl_2_, 1 CaCl_2_, 5.4 KCl, 1 CoCl_2_, 1 BaCl_2_ (250 μM 4-AP), pH adjusted to 7.4 with NaOH. The solution for AP recordings contained (in mM): 140 NaCl, 4 KCl, 1.1 MgCl_2_, 1.8 CaCl_2_, 10 glucose, 10 HEPES, pH adjusted to 7.4 with NaOH. The intracellular recording pipette solution for both Ito and AP contained (in mM): 135 KCl, 4 MgCl_2_, 5 EGTA, 10 HEPES, 10 glucose, 5 Na_2_ATP, 3-phosphocreatine, pH adjusted to 7.2 with KOH.

### Surface electrocardiogram recordings

Mice were anesthetized with sodium pentobarbital (60 mg/kg). Thirty two-gauge needle steel electrodes were inserted subcutaneously, according to a standard three-lead ECG scheme. Acquisition was performed during 8–10 min following anesthesia and data collected for 8–15 consecutive min at a rate of 4 kHz by using a ML136 Animal Bio amplifier. Parameters for each individual animal were calculated from 40 to 45 consecutive traces (LabChart 47.0 Pro, ADInstruments, UK). QTc interval was calculated using a modified Bazzett’z correction formula [QTc = QT/(RR/100)^1/2^ (29)].

### Statistics

All parameters were analyzed by a one-way ANOVA, followed by Newman-Keuls *post hoc* test. Statistical significance was set at *p* < 0.05.

## Results

### Plasma biochemistry and cardiac weight and lipid content

As summarized in Table 1, HFD animals displayed a 40% increase BW, compared to control animals (*p* < 0.001). Heart weight was increased approx. about 7% in HFD mice (*p* < 0.05), although cardiac TGs were not different between control and HFD groups. Plasma insulin (*p* < 0.001) and leptin (*p* < 0.05) were also increased by HFD treatment.

**Table 1:** **Effect of 32-week HFD on body and cardiac weight, cardiac triglyceride content, and insulin and leptin plasma concentration**.

	BW (g)	Heart weight (mg)	Cardiac triglycerides (mg/g tissue)	Plasma insulin (μg/l)	Plasma leptin (ng/ml)
Control	33.9 ± 0.6	160.4 ± 3.1	33.5 ± 3.6	1.9 ± 0.2	12.8 ± 1.9
HFD	47.1 ± 1.1***	175.2 ± 6.1*	27.8 ± 2.6	6.0 ± 0.7***	30.2 ± 4.5*

*p < 0.05; ***p < 0.001 (Newman–Keuls’s test).

### Enzymatic activities involved in mitochondrial uptake of fatty-acids and pyruvate appear to be up- and down-regulated, respectively, in HFD mice

**Figure 1:**
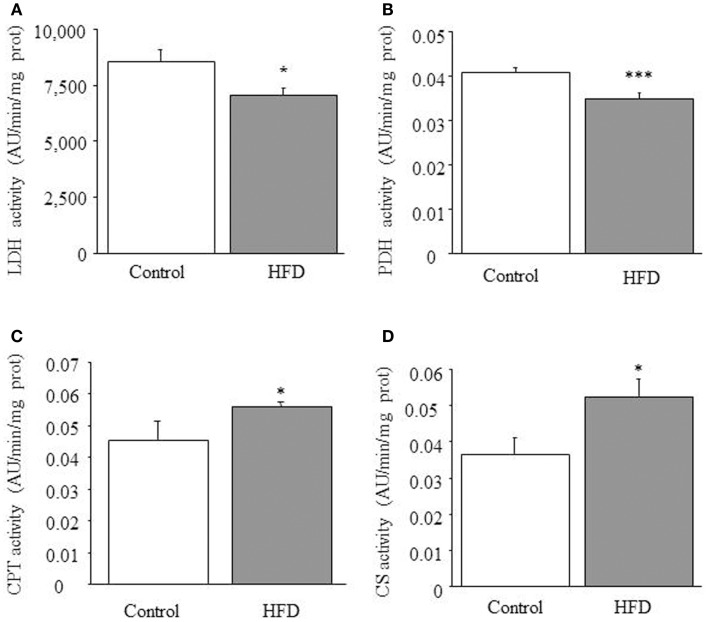
**Enzymatic activities**. Effect of HFD on cardiac lactate dehydrogenase (LDH) **(A)**, piruvate dehydrogenase (PDH) **(B)**, carnitine-palmitoyltransferase (CPT) **(C)**, and citrate synthase (CS) **(D)** activities. Results are expressed as mean ± SEM. Statistical analyses were performed with one-way ANOVA followed by a Newman-Keuls test. *p < 0.05, ***p < 0.001, compared with the control group.

We observed that both lactate dehydrogenase [*F*_(1,21)_ = 5.717; *p* < 0.05; Figure 1A] and pyruvate dehydrogenase activities [F_(1,8)_ = 12.479; *p* < 0.001; Figure 1B] were reduced in HFD mice. This suggests that the contribution of lactate and pyruvate to cardiac energetics would be reduced in HFD mice. Moreover, as previously reported (9), 1-way ANOVA revealed an increase of total CPT activity in HFD mice [*F*_(1,18)_ = 15,417; *p* < 0.05; Figure 1C], suggesting that mitochondrial uptake of FA is up-regulated by HFD treatment.

### Citrate synthase activity and mitochondrial uncoupling

**Figure 2:**
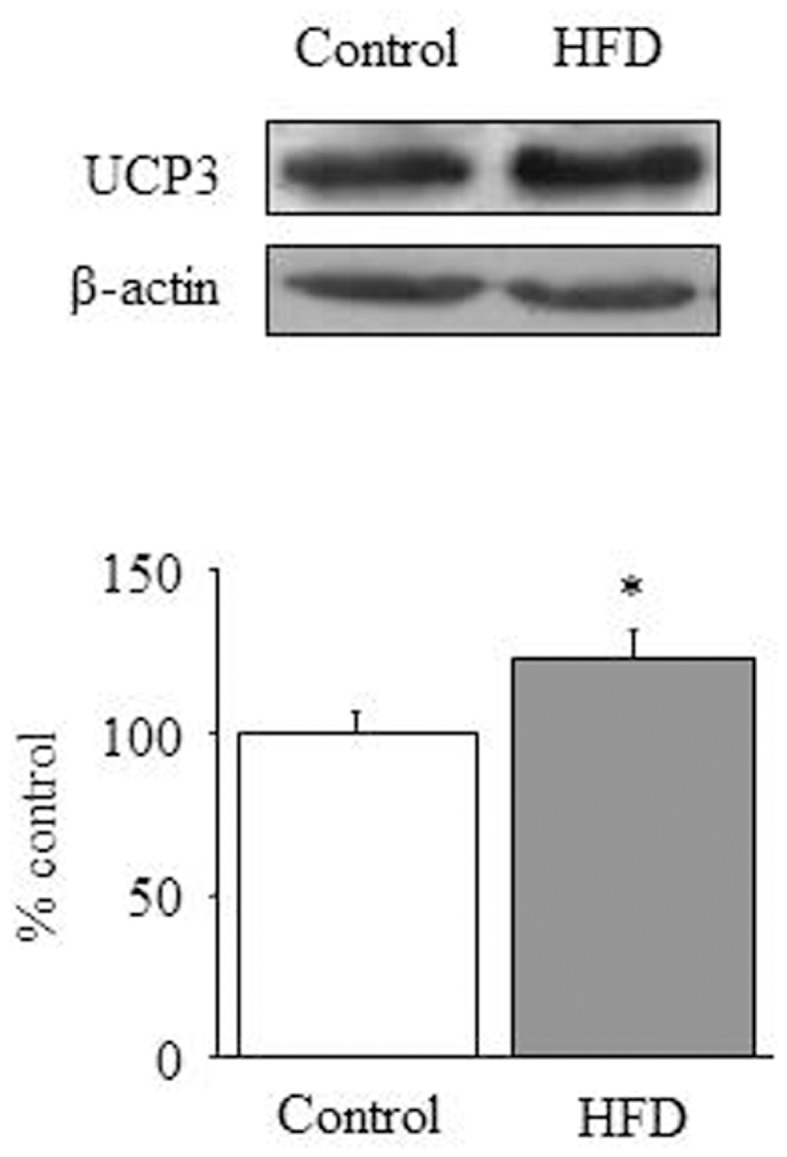
**Mitochondrial uncoupling**. Effect of a HFD on protein expression of cardiac UCP3. Diagram bars show the result of densitometric analysis of UCP3 immunoblots, expressed as percentage of UCP3 in the control group. Data are presented as mean ± SEM. *p < 0.05 compared with the control group.

The ability of cardiac mitochondria to incorporate acetyl-CoA to the tricarboxylic acid cycle can be estimated by measuring CS activity, which accounts for the condensation of acetyl-CoA and oxaloacetic acid to yield citric acid. Figure 1D illustrates the effect of HFD on CS activity, which was significantly increased in HFD mice [1-ANOVA *F*_(1,10)_ = 5,349; *p* < 0.05]. This up-regulation corresponds to almost 30% increase of CS activity. We determined the amount of UCP1, UCP2 (data not shown), and UCP3 and only UCP3 (Figure 2) appeared to be up-regulated in HFD mice [1-ANOVA *F*_(1,12)_ = 5,327; *p* < 0.05].

### Redox status of cardiac tissue after HFD treatment

**Figure 3:**
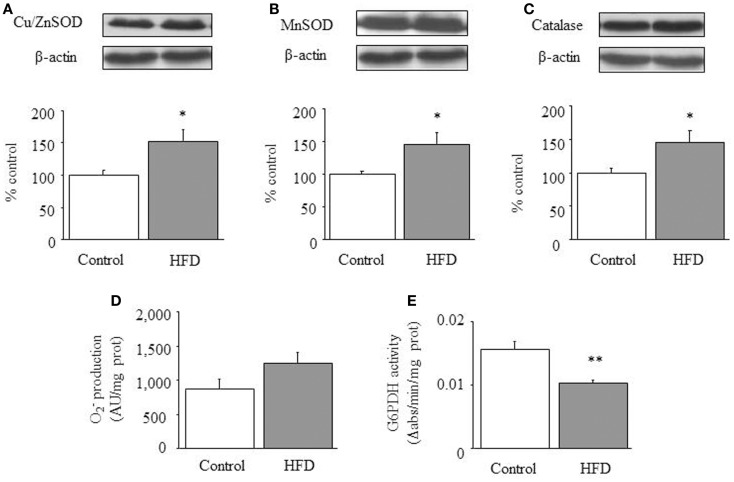
**Redox status in cardiac tissue**. Effect of a HFD on protein expression of cardiac Cu/Zn-SOD **(A)**, Mn-SOD **(B)** and catalase **(C)**, superoxide anion ($O_{2}^{-}$) production **(D)**, and glucose-6-phosphate dehydrogenase activity **(E)**. Diagram bars show the result of densitometric analysis of immunoblots, expressed as percentage of Cu/Zn-SOD, Mn-SOD, and catalase in the control group. Results are presented as mean ± SEM of 6–8 animals/group. *p < 0.05, **p < 0.01, ***p < 0.001 compared with the control group.

Cu/Zn-SOD, Mn-SOD, and catalase were increased in the HFD group [1-ANOVA *F*_(1,11)_ = 5.593, *p* < 0.05; 1-ANOVA *F*_(1,10)_ = 5.017, *p* < 0.05; 1-ANOVA *F*_(1,10)_ = 5.141, *p* < 0.05 respectively; see Figures 3A–C. (Total glutathione)/(Oxidized glutathione) ratios were not different between control (7.69 ± 1.21) and HFD animals (7.76 ± 1.69). $O_{2}^{-}$ production appeared to be slightly increased (*p* = 0.08) in HFD mice (Figure 3D). Finally, we observed a reduction of glucose-6-phosphate dehydrogenase activity (Figure 3E). This enzyme is a main source of NADPH, a co-factor necessary for maintaining redox equilibrium.

### Cellular electrophysiology

**Figure 4:**
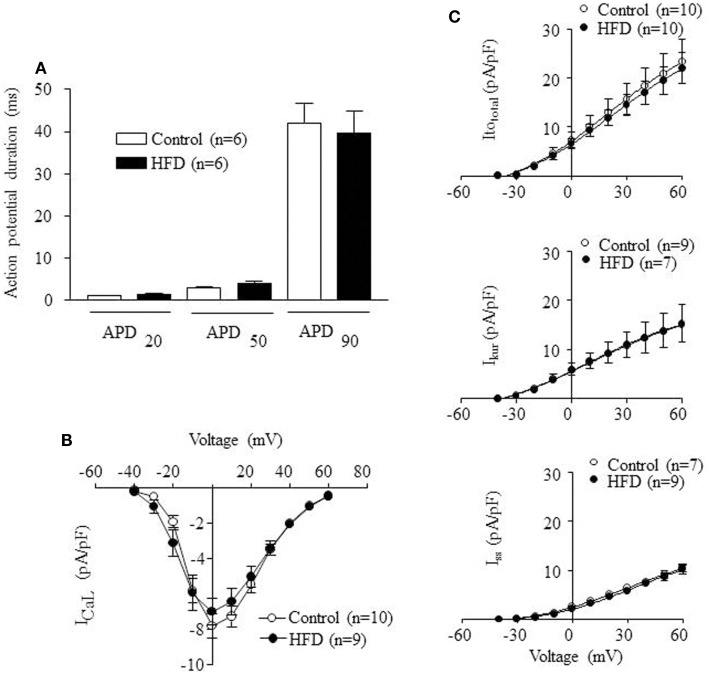
**Electrophysiological properties of ventricular adult myocytes isolated from mice fed with control (open bars) or high-fat diet (HFD) (solid bars) during 32 weeks**. **(A)** Mean action potential duration (APD) values measured at 20, 50, and 90% of repolarization (APD20, APD50, and APD90) in adult myocytes isolated from control and HFD mice **(B)** Current density-voltage relation of l-type calcium current (*I*_CaL_) obtained in adult myocytes isolated from control and HFD mice. **(C)** Current density-voltage relations of three different K^+^ currents obtained in adult myocytes isolated from control and HFD mice: transient outward K^+^ current (Ito_total_), the ultra-rapid delayed rectifier K^+^ current (*I*_kur_), and the steady-state outward K^+^ current (*I*_ss_). Statistical analyses were performed using unpaired Student’s *t*-test.

To analyze the influence of HFD in the cellular electrophysiological properties of ventricular myocytes, patch-clamp experiments were carried-out. Figure 4A shows that mean APD values, measured at 20, 50, and 90% repolarization, were identical in control and HFD ventricular myocytes. Figure 4B shows current density-voltage relationship for *I*_CaL_ obtained in myocytes isolated from control and HFD mice. The maximum value of *I*_CaL_ density was −7.8 ± 0.7 pA/pF at 0 mV (*n* = 10) for control and −7.0 ± 0.7 pA/pF at 0 mV (*n* = 9) for HFD mice. Figure 4C illustrates current density-voltage relationships for *K*^+^ currents (Ito_total_, *I*_kur_, and *I*_ss_). Mean density values and voltage-dependence were similar between control and HFD myocytes. Ito_total_ at +60 mV was 23.4 ± 4.6 *p*A/*p*F (*n* = 10) for control and 22.0 ± 3.1 pA/pF (*n* = 10) for HFD. *I*_kur_ at +60 mV was 15.1 ± 4.2 pA/pF (*n* = 9) for control and 15.3 ± 3.6 pA/pF (*n* = 7) for HFD. *I*_ss_ at +60 mV was 10.5 ± 0.9 *p*A/*p*F (*n* = 9) for control and 10.1 ± 0.9 (*n* = 7) for HFD. Taken together these data indicate that HFD did not induce changes in the cellular electrophysiological properties of ventricular myocytes.

### HFD mice exhibited similar electrocardiographic recordings than control animals

**Figure 5:**
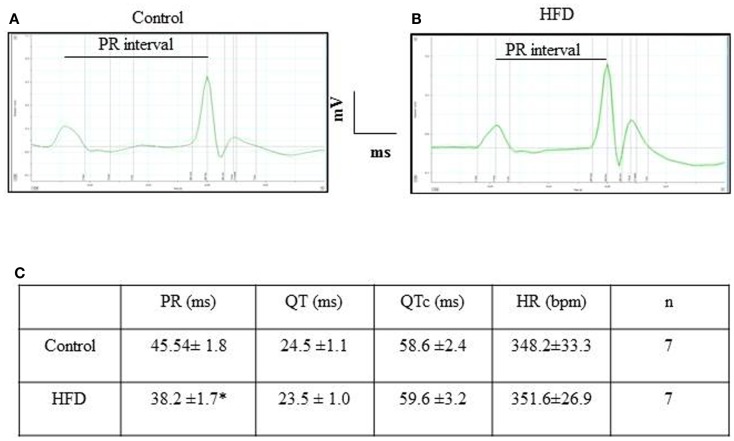
**Electrocardiogram recordings and interval values obtained in control and HFD mice**. Typical surface ECG recording obtained in control **(A)** and HFD mice **(B)**. Intervals duration and heart rate appear summarized in table **(C)**. *p < 0.05, compared to the control group. HR, heart rate.

Figure 5 illustrates typical examples of ECG recordings in control and HFD mice. Heart rates were similar between groups. In addition, QRS complexes, QT, and T wave duration were similar between groups, reflecting a normal velocity of ventricular depolarization/repolarization. PR intervals were slightly shorter (*p* < 0.05) in HFD than in control animals.

## Discussion

This study shows that long-term HFD activates metabolic pathways in cardiac tissue that would (i) promote β-oxidation to the detriment of carbohydrate catabolism, and (ii) prevent cardiac steatosis. This is striking inasmuch as under our experimental conditions both adipose and hepatic tissues undergo pathological alterations of energy metabolism leading to obesity and hepatic steatosis The interest of our animal model (long-term treatment with a relatively mild, 45% kcal from fat, HFD from the early adolescence) deals with the fact that HFD mice display diabetes, obesity, and hyperleptinemia together with a generalized state of leptin resistance that does not involve cardiac tissue (10). This is a very relevant issue because the above mentioned study evidenced that ectopic deposition of lipids in the liver of obese mice is coincident with the development of leptin resistance in this organ. In fact, many authors have reported that HFD causes cardiac steatosis in genetically engineered models of obesity dealing with either generalized leptin resistance or aleptinemia (15, 30–32). Thus, the current data would further support the concept that a particular responsiveness of cardiac tissue to hyperleptinemia might account for the adaptive metabolic remodeling triggered by HFD. Otherwise, although a systematic analysis of heart morphology has not been carried-out, cardiac hypertrophy seems to be absent (cardiac weight appears to be only slightly increased in HFD mice, but appears to be decreased when comparison is made between cardiac weight/BW ratios). Moreover, we have observed that cardiac electrophysiology remains mostly unaffected suggesting that 45% HFD treatment are less detrimental for the heart than for other tissues such as the liver (10) or, even, the central nervous system (33, 34).

In our previous study we had characterized the influence of HFD on the kinetic characteristics of cardiac CPT and we had found that DIO reduces the inhibitory effect of malony-CoA on CPT activity by a mechanism involving the Akt signaling pathway (9). This former study strongly supports the concept that cardiac metabolism adapts to HFD by increasing mitochondrial uptake and further oxidation of FA. In the current study we have evaluated enzymatic activities involved in glucose metabolism and we have identified a decrease of both lactate dehydrogenase and pyruvate dehydrogenase activities together with a reduction of lactate concentration that suggests a low glycolytic rate as well as a small contribution of pyruvate to mitochondrial energy supply. In addition, the elevated CS activity detected in HFD mice suggests an increased production of citrate, which would act as an allosteric inhibitor of phosphofructokinase, the enzyme recruiting glucose-6-phosphate for the glycolytic pathway (16–18). Moreover, increased citrate production would be coherent with an elevated rate of β-oxidation (18). Otherwise, the activity of glucose-6-phosphate dehydrogenase activity appears to be reduced in HFD mice, suggesting that NADPH levels might be reduced by the dietary treatment. The eventual consequences of a fall of NADPH levels are difficult to be drawn as this co-factor is pivotal to maintain the pool of reduced glutathione for reductive process but it is also the substrate of NADPH-oxidase, a source of $O_{2}^{-}$ and ROS production. In fact, $O_{2}^{-}$ levels appears to be slightly increased in HFD hearts while (total glutathione)/(oxidized glutathione) ratios appear to be unchanged by HFD. Otherwise we have detected an up-regulation of antioxidant enzymes. Taken together, all these data would indicate that cardiac tissue of HFD mice remain mostly unaffected in terms of oxidative stress.

Finally we have also explored the effect of HFD on the amount of the uncoupling proteins UCP1, UCP2, and UCP3. In our study UCP3 is the only UCP that appears to be over-expressed in HFD mice. In a recent study Boudina et al. (35) have reported that UCP3 is up-regulated by HFD. These authors propose that UCP3 could be a negative regulator of cardiac efficiency in using energy derived from FA oxidation, although UCP3 lacks of effect on FA oxidation rates. Otherwise, UCP3 might act as a proton leaker and might also facilitate the translocation of FA carboxylate anions out of the mitochondrial matrix. Because carboxylates re-enter the mitochondria as neutral (protonated) species (36), the up-regulation of UCP3 might generate a futile cycling of FA that would contribute to reduce ATP synthesis. In this context, our results would suggest that HFD hearts might display poor energy efficiency. In fact ATP levels appear to be decreased in HFD hearts (0.25 ± 0.03 in HFD vs. 0.40 ± 0.05 nmol/mg protein in control animals; *p* < 0.05; results not shown), although this data is of difficult interpretation in absence of AMP and/or creatinphosphate values. From our data we cannot draw the mechanism that account for UCP3 up-regulation, but we hypothesize that hyperleptinemia might play a role. Interestingly, the former study by Boudina et al. (35) has evidenced that the energy-wasting function attributed to UCP3 is not perceptible in aleptinemic mice, and leptin has been shown to induce UCP3 in different tissues (37, 38). Our data suggest that UCP3 up-regulation would be integral to the adaptive response to HFD/hyperleptinemia, that would contribute to spare ectopic lipid deposit even at expense of a loss of energy efficiency.

Although functional studies are necessary to characterize cardiac function in DIO mice, our data suggest that HFD trigger an efficient metabolic adaptation, able to avoid ectopic accumulation of triglycerides and to limit lipotoxic damage (31), which include the preservation of cardiac electrophysiological properties of the major ionic currents, as already reported to occur in DIO rats (39). K^+^-current remodeling has been observed in metabolic cardiomyopathy induced by PPARα overexpression, supporting the concept that myocardial metabolism and cardiac function are closely linked (40). The shortening of PR intervals detected in the current study is compatible with the existence of accessory atrio-ventricular pathways and suggests that, in spite of a normal behavior of ventricular K^+^-channels, conduction abnormalities might be incipient. To our knowledge, this is the first study showing that a dietary treatment is able to evoke this kind of conduction alteration. Interestingly, this kind of accessory pathways is present at birth in humans (41) and normally disappears few weeks after birth. Nevertheless, they appear to be preserved in adult hearts lacking normal AMPK activity (42). We have previously reported that the active form of AMPK is less abundant in DIO than in control hearts (9) and we speculate now that a HFD treatment from early adolescence might induce *de novo* development of cardiac accessory pathways or, alternatively, to delay the natural degeneration of perinatal atrio-ventricular connections. It has to be noted that ECG recordings were performed in anesthetized animals and, therefore, eventual effects of the anesthesia cannot be discarded.

In conclusion, all these results indicate that metabolic remodeling evoked by HFD appears to be insufficient to affect cardiomyocyte electrical properties, but could trigger incipient functional disorders.

## Conflict of Interest Statement

The authors declare that the research was conducted in the absence of any commercial or financial relationships that could be construed as a potential conflict of interest.
